# Reduced Child-Oriented Face Mirroring Brain Responses in Mothers With Opioid Use Disorder: An Exploratory Study

**DOI:** 10.3389/fpsyg.2021.770093

**Published:** 2022-02-04

**Authors:** James E. Swain, S. Shaun Ho

**Affiliations:** ^1^Department of Psychiatry and Behavioral Health, Renaissance School of Medicine, Stony Brook University, Stony Brook, NY, United States; ^2^Department of Psychology, Stony Brook University, Stony Brook, NY, United States; ^3^Department of Obstetrics, Gynecology, and Reproductive Medicine, Renaissance School of Medicine, Stony Brook University, Stony Brook, NY, United States; ^4^Program in Public Health, Renaissance School of Medicine, Stony Brook University, Stony Brook, NY, United States; ^5^Department of Psychiatry and Psychology, Center for Human Growth and Development, University of Michigan, Ann Arbor, MI, United States

**Keywords:** opioid, maternal behavior neurocircuit, face mirroring, intersubjectivity, empathy, supplementary motor area (SMA), magnetic resonance imaging (MRI), ventral pallidum (VP)

## Abstract

While the prevalence of opioid use disorder (OUD) among pregnant women has multiplied in the United States in the last decade, buprenorphine treatment (BT) for peripartum women with OUD has been administered to reduce risks of repeated cycles of craving and withdrawal. However, the maternal behavior and bonding in mothers with OUD may be altered as the underlying maternal behavior neurocircuit (MBN) is opioid sensitive. In the regulation of rodent maternal behaviors such as licking and grooming, a series of opioid-sensitive brain regions are functionally connected, including the ventral pallidum (VP). In humans, these brain regions, interact with the supplementary motor area (SMA) to regulate maternal behaviors and are functionally dysregulated by opioids. It is unclear how these brain regions respond to the emotions of their child for mothers receiving BT. In this functional magnetic resonance imaging (fMRI) pilot study in 22 mothers within the first postpartum year, including six mothers receiving BT and 16 non-OUD mothers as a comparison group (CG), we devised a child face mirroring task in fMRI settings to assess maternal responses to pictures of facial expressions of own child and an unknown child in an empathic mirroring condition (Join) and a non-mirroring observation condition (Observe). In each condition, faces of neutral, ambiguous, distressed, and joyful expressions of each child were repeatedly displayed in a random order. The response of SMA during empathic mirroring (Join) vs. non-mirroring (Observe) of own child was reduced among BT/OUD vs. CG. Within MBN, the left VP, critical for parental sensitivity, had a similar deficit. This study outlines potential mechanisms for investigating the risks of deficits in the neural responses to actual maternal sensitivity and parenting behavior in mothers with OUD, and potential targets for interventions that reduce stress and augment maternal behavior and child outcome.

## Introduction

Every day in the United States, approximately 200 people die after overdosing on opioids (CDC/NCHS, 2021). The incidence of pregnant women with opioid use disorder (OUD) quadruped from 1999 to 2014 (from 1.5/1,000 delivery hospitalizations to 6.5) (Haight et al., 2018). In this epidemic, 2.5% of pregnant women use opioids chronically (Krans and Patrick, 2016) such that about 100,000 postpartum women and their families are afflicted with OUD every year. However, pregnant women with OUD may receive “gold standard” buprenorphine treatment (BT) for withdrawal (Jones et al., 2012; Nanda et al., 2015; Krans et al., 2016; Rosenthal et al., 2016; Zedler et al., 2016). Buprenorphine is a semisynthetic morphine-derived opioid used to treat OUD and chronic pain with very high affinity for the μ-receptor as a partial agonist and high affinity for the κ-receptor as an antagonist. Despite withdrawal reduction with BT, pregnant and postpartum women remain at high risk for problems, for which treatment is lacking. Indeed, relapse is common, with comorbid high stress, depression, polysubstance use, and maladaptive parenting behaviors (Rutherford et al., 2011; Rutherford and Mayes, 2017; Swain and Ho, 2019; Swain et al., 2019) risking child maltreatment and costly foster care utilization (Conway et al., 2006). Thus far in humans, however, there is still little research on mother and child bonding and health with buprenorphine treatment for OUD (Salihu et al., 2019).

Of additional concern to mothers with OUD, exogenous opioid-induced deficits have been shown for maternal behaviors in animal models (Bridges and Grimm, 1982; Grimm and Bridges, 1983; Slamberova et al., 2001). At least in part, these effects appear to be mediated by the activation of μ-opioid receptors in the hypothalamic (HYP) medial preoptic area (mPOA) (Rubin and Bridges, 1984; Mann et al., 1991; Stafisso-Sandoz et al., 1998). As part of the opioid-sensitive brain, the mPOA regulates a series of neurocircuits in the regulation of many salient behavioral outputs (Berridge and Kringelbach, 2015). For rodent maternal behaviors, the HYP normally activates the nucleus accumbens (NAc) and ventral pallidum (VP) (Numan and Young, 2016). Human mothers have a homologous and adaptable maternal behavior neurocircuit (MBN) as outlined by functional magnetic resonance imaging (fMRI) and responses to infant stimuli (Swain et al., 2007, 2019; Swain and Lorberbaum, 2008; Kim et al., 2016; Swain and Ho, 2017).

**FIGURE 1:**
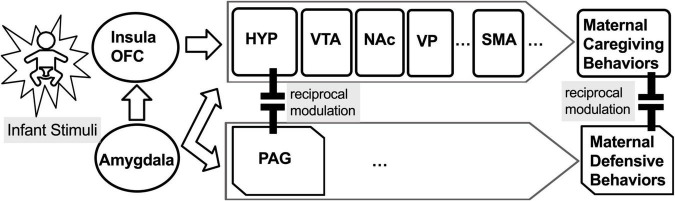
The maternal behavior neurocircuit (MBN) is comprised of two reciprocally inhibiting subsystems for: (1) maternal care, mediated by the medial preoptic area (mPOA) of hypothalamus (HYP), ventral tegmental area (VTA), nucleus accumbens (NAc), and ventral pallidum (VP), which is functionally connected to the supplementary motor area (SMA), and (2) maternal defense, mediated by periaqueductal gray (PAG). These opposing subsystems are regulated by the amygdala (AMY), insula, and orbitofrontal cortex (OFC).

The MBN regulates mother–infant bonding, balances sensitive caring vs. aggressive defensive maternal behaviors in humans and other mammals, and adapts to a variety of circumstances (Swain and Ho, 2019). In addition to the mPOA and VP, many other MBN areas are sensitive to exogenous opioids (Wallin et al., 2021), including the NAc, VTA for parental care, and PAG for parental defensive behavior. In brain models extended to include substance use, the VP has previously been proposed as a common pathway for drug seeking initiated by stress, drug-associated cues, or the drug itself (Kalivas and Volkow, 2005). In fact, normal function in the VP is extremely important for discriminating between natural and exogenous drug-related rewards (Root et al., 2015). In preclinical animal models, natural offspring stimuli cause maternal brain activation of the NAc-VP circuit (*via* dopamine and oxytocin) to facilitate selective offspring recognition, behavioral reactivity, and lasting social attraction. This can occur when NAc-GABAergic efferents to the VP are suppressed (*via* cortical dopamine-induced disinhibition), releasing the VP from NAc inhibitory control and enhancing VP response to pup stimuli (Hansen et al., 1993; Numan and Insel, 2003; Champagne et al., 2004; Numan, 2007; Ikemoto, 2010). The MBN is modulated by the opioid-sensitive extended amygdala, including the bed nucleus of the stria terminalis (Klampfl and Bosch, 2019), insula, and orbitofrontal cortex (Gholampour et al., 2020) with connections to motor cortical regions for maternal behavioral output (Figure 1), such as the supplementary motor area (SMA) (Zhang et al., 2012). The SMA is activated by infant crying sounds, for which picking up, holding, and talking to their infants are behaviors common to mothers across multiple cultures (Bornstein et al., 2017). Thus far, however, there is little research on mothers with buprenorphine treatment for OUD (Salihu et al., 2019), and just a few studies recently reviewed on the underlying MBN among mothers with OUD (Swain and Ho, 2021).

In our first report, BT mothers with OUD compared with a control group (CG) showed greater HYP and PAG responses to own vs. other baby-cry and differential functional connectivity between the HYP and PAG associated with parenting stress, suggesting that BT may dysregulate the normal balance between maternal caregiving and defensive/aggressive circuits (Swain et al., 2019). In another study of the same cohort, BT vs. CG differences in resting-state functional connectivity (rs-FC) between the PAG and HYP were studied at 1 month (T1) and 4 months postpartum (T2) (Swain and Ho, 2019). The authors found that BT mothers differed from CG mothers in PAG-dependent rs-FC with the HYP, amygdala, insula, and other brain regions that regulate caring at T1, with many of these differences not evident at T2. Furthermore, the authors also found that the PAG-dependent rs-FCs were related to maternal bonding problems as evidenced by the fact that “rejection and pathological anger” subscale of the Postpartum Bonding Questionnaire (PBQ) at T2 was associated with the increases from T1 to T2 in PAG-dependent rs-FC with the HYP and amygdala. This suggests that possible maternal bonding problems for mothers with BT OUD in the early postpartum may be linked to connectivity differences between specific care and defense maternal brain circuits, which may also be modulated by buprenorphine treatment. More work is required to elucidate how the MBN regulates specific parenting behaviors in OUD mothers such as maternal sensitivity that relate to infant outcome.

Parent–child interactions involving sensitively sharing joy and coping with distress are crucial for child development (Swain et al., 2017). Parental intersubjectivity has been identified as a key resilience factor against the adverse effects of parental stress and depressive moods on parent–child relationships (Camoirano, 2017; Bernard et al., 2018). Intersubjectivity is defined here as the understanding of the internal models of self and others, intentions, and feelings underlying overt behaviors. Parental intersubjectivity enables a parent to feel what the inner experience of a child *is like*, without diminishing the distinction between the inner experiences of the parent and the child. Parental intersubjectivity is embedded in several parenting-related constructs, such as parental sensitivity (Ainsworth et al., 1978; Bernard et al., 2013), parental reflective functioning (Fonagy et al., 1991; Slade, 2005), parental empathic attunement (Rowe and MacIsaac, 2004), and parental embodied mentalizing (Shai and Belsky, 2011). These constructs commonly point to the capacity of a parent to rely on dyadic interactions to understand the child and provide sensitive care to foster healthy development.

A key attribute underlying intersubjectivity is face mirroring, i.e., spontaneous mimicry or voluntary imitation of the facial expressions or manual gestures of others. The rudimentary capacity of intersubjectivity is innate (Trevarthen and Aitken, 2001). Indeed, infants can spontaneously mimic facial expressions soon after birth (Meltzoff and Moore, 1977). While mothers with secure parent–child bonding show greater child-oriented face mirroring (Kim et al., 2014), unfortunately, maternal intersubjectivity may be impaired in mothers exposed to excessive parenting stress (Shai et al., 2017), interpersonal violence (Dayton et al., 2016), or depressive mood disorders (Bernard et al., 2018). We have previously demonstrated that a parenting intervention delivered a few years postpartum reduced parenting stress with associated increases in parent–child intersubjective function in the MBN (Ho et al., 2020). Specifically, we found that SMA and other MBN regions were differentially activated during the condition in which the mothers empathically mirrored the facial expressions and emotions of the child. In this study, we contributed data from a pilot project on mothers with BT mothers with OUD using an empathic mirroring fMRI task described below. We hypothesized that the MBN required for mothers to empathically mirror the emotions of their child in infant-oriented sensitive behaviors may be altered for mothers under the stressful conditions of OUD receiving BT.

## Materials and Methods

The research reported in this study was approved by the Institutional Review Board (IRB) at the University of Michigan, Ann Arbor, MI, United States. All research was performed in accordance with relevant IRB guidelines and regulations.

### Participants

All participants (*N* = 22) were recruited from University of Michigan Health System. There were six participants in the buprenorphine replacement treatment group (BT) and 16 participants in the comparison group (CG) who underwent the fMRI task within 1 year postpartum. The participants in BT and CG groups were not different in age [BT: *M* = 30.67, s.e. = 2.68; CG: *M* = 29.63, s.e. = 1.64, *F*_(1,20)_ = 0.11, MS_error_ = 43.154, *p* = 0.74], the age of their youngest child [BT: *M* = 0.25, s.e. = 0.06; CG: *M* = 0.20, s.e. = 0.04, *F*_(1,20)_ = 2.47, MS_error_ = 0.024, *p* = 0.13], and the number of offspring [BT: *M* = 1.83, s.e. = 0.24; CG: *M* = 1.50, s.e. = 0.15, *F*_(1,20)_ = 1.42, MS_error_ = 0.34, *p* = 0.25] (refer to Table 1 for other demographics). The BT OUD mothers were monitored with urine screens and interview as part of clinical care during pregnancy, such that the only exogenous opioid was prescribed buprenorphine. As recorded every 2 weeks postpartum and during our study, it was 12.67 ± 1.63 mg (mean ± SD) daily with all mothers stabilized between 12 and 16 mg daily. We have reported fMRI studies using different tasks completed in the same cohort including a baby-cry task (Swain et al., 2019) and resting-state task (Swain and Ho, 2019).

**TABLE 1:** Demographics.

	BT	CG
**Age**		
Mean	30.67	29.63
s.e.	2.68	1.64
**Infant age**		
Mean	0.25	0.20
s.e.	0.06	0.04
**Number of child**		
Mean	1.83	1.50
s.e.	0.24	0.15
**Race**		
European American	5	12
African American	0	3
Native American	1	0
Bi-racial	0	1
**Socioeconomic status**		
Low	4	11
Middle	2	5

*BT, buprenorphine treatment for opioid use disorder; CG, comparison group.*

### Child Face Mirroring Task

**FIGURE 2:**
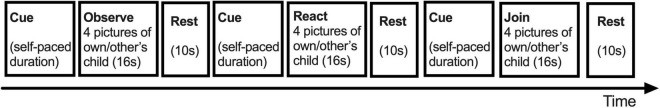
The design of child face mirroring task [adapted from Ho et al. (2020)]. Note that the task order in this figure did not represent the actual order.

In Child Face Mirroring Task (CFMT), as described previously (Ho et al., 2020) and illustrated in Figure 2, the participants were presented repeatedly with the same pictures of their own child and of an unknown child in three task conditions, namely, Observe, React, and Join. By design, the Observe task should elicit the unresponsive observation of face-like visual objects of participants; the React task should elicit the usual, voluntary responses of participants to the presented child, and the Join task should elicit the empathic mirroring of participants of the presented child. The React condition was designed for a pre- and post-treatment study (Ho et al., 2020) and thus was not included in the analysis of this study. The task instructions, design, and stimuli have been described elsewhere (Ho et al., 2020).

### Magnetic Resonance Imaging Procedures

The magnetic resonance imaging (MRI) procedures, image acquisition, and data preprocessing have been described elsewhere (Ho et al., 2020). No head movements.

#### First-Level Analysis

Following image preprocessing described elsewhere (Ho et al., 2020), we constructed a first-level fixed effect general linear model (GLM) to examine condition-dependent responses. The first-level model consisted of a matrix of regressors modeling six trial types (3 Tasks × 2 Child Identities: Observe Own, React Own, and Join Own and Observe Other’s, React Other’s, and Join Other’s Child), in addition to a regressor for Cue periods (seven regressors total). In this study, we focused on the contrast of the Join vs. Observe contrast of Own Child. Handedness and possible functional lateralization of brain function in the participants were not considered in this study.

#### Second-Level Analysis

Due to the small sample size, we focused on one contrast of interest, i.e., Join vs. Observe of the Face of Own Child, pooling across facial expressions in this study. This contrast of interest from the first level GLMs was submitted to a second-level random effect GLM, testing BT vs. CG effects on several regions of interest (ROIs), with Bonferroni family-wise small volume corrections (s.v.c.) in each ROI. The ROIs were identified as the subcortical regions known to modulate maternal behaviors (Numan and Woodside, 2010; Swain and Ho, 2017, 2019), with their masks derived from the wfu_pickatlas toolbox (Maldjian et al., 2003), including amygdala [as defined in wfu_pickatlas’ AAL domain (Tzourio-Mazoyer et al., 2002)], periaqueductal gray (PAG) (an 8 mm × 6 mm × 8 mm box centered at [0, −28, −12] in MNI coordinates), hypothalamus (as defined in wfu_pickatlas’ TD Brodmann areas+ domain; Maldjian et al., 2003), midbrain (as defined in wfu_pickatlas’ TD Lobes domain; Maldjian et al., 2003), nucleus accumbens (NAc) (a 18 mm × 8 mm × 10 mm box centered at [0, 10, −14] in MNI coordinates), and pallidum (as defined in AAL; Tzourio-Mazoyer et al., 2002). In addition, as from an independent sample of late postpartum mothers (*N* = 45), reported previously (Ho et al., 2020), we found that SMA (MNI coordinates: [0, 6, 58], 76 voxels, *Z* = 5.28, *p* < 0.001 whole-brain corrected) was the only cluster surviving the whole brain family-wise Bonferroni correction in the main effect of the contrast of interest (Join vs. Observe Own Child). We, therefore, selected SMA as another ROI in this study and created a mask defined by this cluster (682 voxels) as found in that independent sample at a threshold of *p* = 0.005, uncorrected.

## Results

### Main Effect of Join Versus Observe of Own Child

**FIGURE 3:**
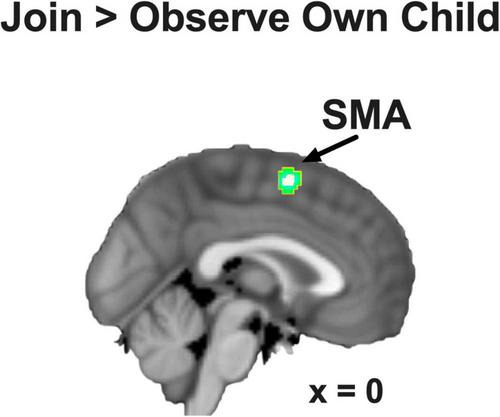
The SMA showed Join > Observe of Own Child differential neural response, pooling across groups. The statistical map is presented with an activation threshold of *p* = 0.005, uncorrected.

We focused on the contrast of Join vs. Observe of Own Child in this study. As hypothesized, we found that, pooling across BT and CG groups, the SMA showed significant Join > Observe differential neural responses in this contrast (MNI coordinates: [0, 2, 58], 65 voxels, *Z* = 3.53, *p* = 0.014 Bonferroni family-wise s.v.c., Figure 3). There were no other ROIs that showed significant differential neural responses in this contrast.

### Buprenorphine Treatment Versus Comparison Group Contrast

**FIGURE 4:**
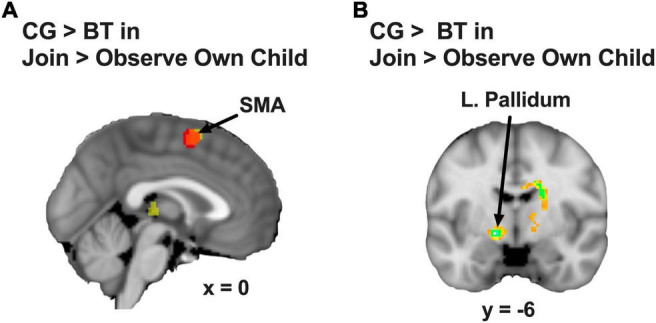
**(A)** The SMA (the red area indicates the overlap with the Figure 2) and **(B)** the left pallidum showed CG > BT group difference in Join > Observe of Own Child differential neural response. The statistical map is presented with an activation threshold of *p* = 0.005, uncorrected.

**FIGURE 5:**
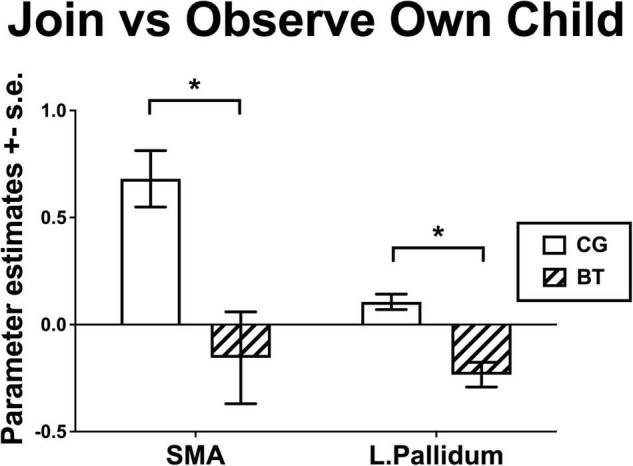
The bar charts for the Join vs. Observe of Own Child differential responses of BT and CG groups (mean ± s.e.) in the SMA and left pallidum. *indicates that significant group difference in the fMRI analysis.

We examined the group differences in the contrast of Join vs. Observe of Own Child. We found that BT showed lesser differential neural responses than CG in the SMA ([2, 8, 60], 222 voxels, *Z* = 3.13, *p* = 0.045 Bonferroni family-wise s.v.c., Figure 4A) and the left pallidum ([−16, −4, −6], 23 voxels, *Z* = 4.26, *p* = 0.001 Bonferroni family-wise s.v.c., Figure 4B). As depicted in the bar chart (Figure 5), in the SMA and left pallidum both, the CG group showed significant Join > Observe differential responses, but the BT group showed Join < Observe differential response. These results suggested that BT mothers may have altered emotional mirroring responses in brain regions important for parenting behaviors.

## Discussion

In the midst of an unprecedented opioid overdose crisis (CDC/NCHS, 2021), many peripartum women with OUD are successfully treated with opioid replacement treatment that reduces withdrawal yet poses potential concerns for the psychology of parenting (Salihu et al., 2019). Animal model research has raised substantial concerns that opioids may disrupt maternal behavior by acting on opioid-sensitive maternal brain circuits including the hypothalamus and VP (Bridges and Grimm, 1982; Grimm and Bridges, 1983; Slamberova et al., 2001). Although allied research in humans has suggested that opioids like buprenorphine might reduce separation distress and offer treatment for some forms of depression (Panksepp and Yovell, 2014; Yovell et al., 2016), there have also been concerns that opioids may usurp healthy parent–infant separation distress and reward circuits that may be critical to mother–infant bonding (Swain et al., 2005). Indeed, “high opioid tone” has been recently discussed as a concern in the development of autism spectrum disorder (Anugu et al., 2021), which is arguably one of several developmental disorders showing impaired intersubjective function (Trevarthen and Aitken, 2001). Thus, although BT is highly effective for reducing withdrawal, intersubjectivity-dependent face mirroring may be adversely influenced. By examining the multifaceted psychosocial effects of BT in the early postpartum on the maternal brain, this article begins to address the potential risks and benefits of buprenorphine beyond the basic relief of withdrawal in OUD.

An emerging human neuroimaging literature supports specific mechanisms at work in mothers with BT/OUD for maternal response to the baby cry and functional connectivity in care and defense brain systems (Swain and Ho, 2021). In this pilot study, we tested the MBN for BT vs. CG group differences in child-oriented face mirroring, a foundational aspect of parental intersubjectivity, that may have long-term consequences for infant development (Feldman, 2012), using the contrast of Join vs. Observe of Own Child in CFMT. Pooling across both groups, the SMA showed significant differential activation in this contrast, replicating the results from an independent sample of healthy mothers scanned at a later postpartum timepoint with the same task (Ho et al., 2020). We also found preliminary effects of BT/OUD on the differential neural responses during child-oriented mirroring, i.e., BT/OUD mothers, as compared to CG, showed altered differential neural responses in the SMA and left VP, an opioid sensitive part of the MBN. While the CG showed Join > Observe differential responses, the BT/OUD group showed little differential response in the SMA and in the opposite direction for Observe > Join differential responses in the VP.

The results suggested that, as related to the comparison group, BT/OUD mothers showed impairments in the own-child-oriented face mirroring responses in brain areas that are critical to maternal intersubjectivity including the VP and SMA. The VP is an important part of the MBN in the regulation of maternal caregiving (Swain et al., 2019) and reward processing in addictions (Kalivas and Volkow, 2005; Root et al., 2015). In animal models, the VP has been demonstrated to be a target of maternal brain motivational output of the NAc (Numan and Woodside, 2010; Numan, 2014) and involved in primate models of cued reaching (Jaeger et al., 1995) and other motivated movements (Hegeman et al., 2016). Among human mothers, the VP has been activated in fMRI studies of mothers observing salient own vs. other baby stimuli (Swain, 2011) and increased for mothers viewing the feeding behavior of their own vs. other children at 2–3 years of age (Noriuchi et al., 2019). Furthermore, VP and SMA responses correlated with maternal caregiving behaviors (Hipwell et al., 2015) and responded in a face mirroring task similar to that used in this article (Ho et al., 2020) for non-OUD mothers. Across continents and cultures, the SMA was highlighted in response to own vs. other baby-cry for infant-oriented preparation for movement and vocalization (Bornstein et al., 2017) and also demonstrated to be important for child-oriented empathy in a parent decision-making fMRI task (Ho et al., 2014). Finally, SMA connectivity to amygdala was reported to be heightened during maternal responses to infant distress according to maternal childhood maltreatment and decreased maternal intrusive behaviors, suggesting the potential for transgenerational adaptations to early life adversity that could include brain responses and infant-oriented behaviors to increase maternal sensitivity (Olsavsky et al., 2021). Perhaps plasticity in the SMA could be a future target for interventions to address maternal health from early childhood maltreatment to OUD such as transcranial magnetic stimulation.

This study on responses to a face mirroring task for mothers with BT OUD is preliminary and with notable limitations. First, an optimal comparison group of OUD mothers without BT is neither feasible nor ethical because of the practical impossibility of recruiting subjects with untreated OUD and the medical imperative to treat the suffering of any subject with OUD, respectively. Thus, our CG mothers were not affected by OUD, related stress, or the possible influence of previous opioid use. Future research may need to adopt approximate controls according to the measures of stress or longitudinal designs in which subjects may be their own controls at different doses and time points. Second, replication with larger sample sizes is needed to confirm these findings and include full characterization of participants with OUD, including the quantity and frequency of all prescription, licit and illicit drug using, cravings, withdrawal, and the gold standard “time-line follow back” interview with calendar prompts and other memory aids to facilitate comprehensive and accurate recall of drug use (Sobell et al., 1988, 1998; Carey, 1997). These data in future studies will allow us to test the assumption of it for a range of critical factors. Indeed, the effects of childhood adverse experiences, sociodemographic factors, and other medical conditions constitute important areas of future research on intersubjective parental function, since we already know that parental stress, poverty, anxiety, and postpartum depression affect the parental brain (Moses-Kolko et al., 2014; Kim et al., 2015; Ho and Swain, 2017; Guo et al., 2018). Currently lacking studies of deficits and resiliencies in addition to possible lateralization in maternal brain function connected with mother–child bonding, parenting behavior and child outcome may contribute insights into the long-term consequences of OUD toward improved prophylaxis and treatment (Moningka et al., 2019).

## Conclusion

Although preliminary, this study probes potential buprenorphine effects on intersubjective child face mirroring responses in mothers affected by OUD. These preliminary results strengthen the hypotheses that specific MBN regions that are required for mothers to empathically mirror the emotions of their child in infant-oriented sensitive behaviors may be altered for mothers with the stressful condition of OUD receiving BT. With replication and converging research on parental interventions that affect the same regions and correlate with inexpensive and convenient questionnaires, it may be possible to maximize intervention effects on specific neural targets for mothers to augment maternal intersubjectivity and reduce transgenerational mental health risks. Perhaps future interventions will be tailored according to neural targets as needed just as other treatments in medicine target-specific physiological systems that may be malfunctioning. This report calls for more attention to parental intersubjectivity and the roles of SMA and VP in the MBN as possible underlying brain mechanisms to better assess opioid-sensitive parental brain functions in the context of parent–child bonding and parenting.

## Data Availability Statement

The raw data supporting the conclusions of this article will be made available by the authors, without undue reservation.

## Ethics Statement

The studies involving human participants were reviewed and approved by the Institutional Review Board of the University of Michigan. The patients/participants provided their written informed consent to participate in this study.

## Author Contributions

JS and SH wrote the manuscript—shared in conceptualization, writing, reviewing, editing grant support, and project administration and approved the submitted version.

## Conflict of Interest

The authors declare that the research was conducted in the absence of any commercial or financial relationships that could be construed as a potential conflict of interest.

## Publisher’s Note

All claims expressed in this article are solely those of the authors and do not necessarily represent those of their affiliated organizations, or those of the publisher, the editors and the reviewers. Any product that may be evaluated in this article, or claim that may be made by its manufacturer, is not guaranteed or endorsed by the publisher.
